# Stigmatization among People Living with HIV/AIDS at the Kumba Health District, Cameroon

**DOI:** 10.1177/2325958219899305

**Published:** 2020-01-07

**Authors:** Thomas Obinchemti Egbe, Cynthia Adanze Nge, Hermann Ngouekam, Etienne Asonganyi, Dickson Shey Nsagha

**Affiliations:** 1 Department of Obstetrics and Gynaecology, Faculty of Health Sciences, University of Buea, Buea, Cameroon; 2 Department of Public Health and Hygiene, Faculty of Health Sciences, University of Buea, Buea, Cameroon; 3 District Hospital Kumba, Southwest Region, Kumba, Cameroon

**Keywords:** human immunodeficiency virus, stigmatization, people living with HIV, Kumba Health District, Cameroon

## Abstract

We determined the level, type of stigma, and risk factors associated with stigmatization of people living with HIV/AIDS (PLWHA) by conducting a cross-sectional study from April to June 2018 in 3 HIV treatment centers in the Kumba Health District (KHD), Cameroon. We reviewed hospital registers, conducted focus group discussions, and administered structured questionnaires. For data analysis, we used the Statistical Package for Social Sciences version 20.0. We recorded a total stigma index score of 59.1. Internal stigma (odds ratio [OR] 2.91; 95% confidence interval [CI]: 1.74-4.98) was common in PLWHA. Also, younger age <30 years (adjusted OR [AOR]: 0.39; 95% CI: 0.17-0.94) was linked with stigma reduction while low level of education (AOR: 1.74; 95% CI: 1.02-2.97) increased the stigma level. HIV-related stigma is pervasive in the lives of PLWHA, with most of them having internal stigmatization. Appropriate health education on HIV will be crucial in reducing stigmatization in the KHD.

**What Do We Already Know about This Topic?**

Findings from 50 countries indicate that roughly 1 in every 8 people living with HIV do not have access to health services because of stigma and discrimination.

**How Does Your Research Contribute to the Field?**

It showed that older people (aged above 30) and persons with just primary level of education were more stigmatized, implying the presence of a knowledge gap. These findings serve as evidence and key indicators in designing and implementing HIV/AIDS antistigma projects in the KHD and Cameroon at large.

**What Are the Implications of Your Research toward Theory, Practice, or Policy?**

Targeting the rural areas where there is a dearth of information regarding HIV due to low education and poverty will be a good approach for stakeholders in reducing stigmatization.

## Introduction

HIV/AIDS is a major cause for concern with an estimated prevalence of 36.7 million HIV-positive persons worldwide in 2016 with a total of 1 million deaths globally.^1^ HIV/AIDS-related stigma as defined by The Joint United Nations Program on HIV and AIDS (UNAIDS) is the process of devaluing people either living with or associated with HIV/AIDS infection.^2^ It has proven to have negative effects on health outcomes such as low adherence to highly active antiretroviral therapy (HAART) due to inconsistency in showing up at treatment centers to pick up their drugs, increased depression, and overall lower quality of life.^3–6^

HIV-related stigma has been associated with a lack of proper information regarding the spread of the disease, fear, and moral judgment from those living with the disease.^7^ Studies have shown discrimination in health-care facilities or the community toward people living with HIV manifested in the form of denial of care, confidentiality breaches, and humiliating attitudes^8,9^ or gossiping from members of the community.^6^ This perceived community discrimination (external) stigma leads to internalized stigma (self-exclusion from social gatherings and public events) and anticipates stigmatizing exposure resulting in adverse health and psychosocial outcomes.^6^ Notwithstanding that, the increasing recognition of the negative effects of HIV-related stigma is associated with a dearth of evidence-based interventions that assess the psychological well-being of people living with HIV/AIDS (PLWHA).^10^

In 2006, the UNAIDS alongside other organizations such as the Global Network of People Living with HIV, the International Community of Women Living with HIV/AIDS, and the International Planned Parenthood Federation drafted a guide that is globally recognized and used by most people in researching this area of study: “The People Living with HIV Stigma Index” is used to ease the measurement of stigma and discrimination in PLWHA in communities and to better understand what they are experiencing.^10^

This tool has not yet been fully utilized in Cameroon, unlike other sub-Saharan countries. Thus, it was one of the main reasons we used it to assess the level of HIV/AIDS-related stigma experienced by 385 PLWHA in 3 HIV treatment centers in the Kumba Health District (KHD), Cameroon. The result obtained from this research study will serve as advocacy material in designing HIV/AIDS antistigma projects.

## Materials and Methods

### Study Design

We conducted an analytical cross-sectional study from April to June 2018.

### Study Area and Duration of Study

The study was conducted in 3 primary health-care facilities in the KHD, authorized by the Cameroon Ministry of Public Health to serve as HIV treatment centers for PLWHA (District Hospital Kumba [DHK], Star Light Association, and Kumba Urban Sub-Divisional Hospital).

### Sampling Method and Sample Size Determination

Purposive sampling was used to choose the 3 HIV treatment centers in the KHD. Probability proportionate to size was used to select the total number of participants in each of the treatment centers, and consecutive sampling was used to recruit participants in each of these facilities.

The sample size was determined using Cochran formula, with a precision rate of 5% and 95% confidence interval (CI).^11^ A minimum of 385 participants were required for the study.

### Study Procedure

All interviews were conducted in a private room, where no one could overhear our discussion. We carried out 4 focus group discussions (FGDs) using an FGD guide, 10 persons (interviewer inclusive) per session, and the responses were audio-recorded and transcribed verbatim following informed consent from the participants.

The PLWHA stigma index questionnaire used to measure the stigma level was divided into 3 broad parts. The first part concerned participant’s sociodemographic characteristics (age, sex, marital status, and length of time on treatment).

The second part of the questionnaire was subdivided into 3 parts:

- participant’s experiences of discrimination by others within the last 12 months;
- participant’s access to work, health, and education services; and
- aspect of internal or self-stigma.

The last part of the questionnaire focused on the participant’s testing, diagnosis, disclosure, and confidentiality.

The questions were mostly closed-ended questions with 4 choices (never, once, a few times, or often). Both FGDs and questionnaires were used in a mixed manner to appropriately capture data to better understand the psychological well-being of the participants. The interview sessions and FGDs took about 1 hour and were conducted in English and Pidgin English. The FGDs were scheduled by the heads of the various HIV treatment centers where data were collected, and the participants were randomly selected after signing the informed consent form. The FGD guide was prepared with the help of the heads of the HIV treatment center in the DHK, which is the biggest HIV treatment center in the KHD (Supplementary Appendix 1). The FGD sessions were very useful, as some of the participants opened up and spoke about their personal experiences, which they didn’t feel comfortable answering in the questionnaires.

### Data Management and Analysis

Participant questionnaires were compiled into a booklet and kept safely, and results were keyed into MS Excel 2013 on a password-protected computer. The data were analyzed using Statistical Package for Social Sciences (SPSS) version 20.0. Continuous variables such as age were expressed as percentage, and logistic regression was computed to identify factors significantly associated with stigma. To get the stigma level, the score of each participant was calculated for 7 (external stigma) and 13 (internal stigma) questions. These scores were calculated by dividing the total of the individual scores by the total sample size. The stigma index, estimated on a scale of 1 to 100, was obtained by multiplying the scores by a unit value of 20. The stigma was categorized as stigmatized and nonstigmatized.

### Ethical Approval and Informed Consent

This study was approved by the institutional review board of the Faculty of Health Sciences, University of Buea, Cameroon (Ref. No. 2018/205/UB/SG/IRB/FHS). All participants provided written informed consent prior to enrollment into the study.

## Results

A total of 385 participants completed the study, and 87.3% were from a single center. The majority of the participants were female (73.2%), and about two-third fell between the 31- and 50-year age bracket (66.6%). About one-quarter (23.9%) had postsecondary education, and more than two-thirds were married (70.6%). The length of time since diagnosis of HIV was ≤5 years in 76.4%, and 76.1% had 1 to 5 children. Furthermore, 63.1% of the participants were farmers. Most (93%) of the study participants did not belong to any support group (Table 1). As regards self-stigmatization, 44.4% of the respondents felt ashamed because of their HIV status, 29.9% of them exhibited self-guilt, and 31.9% blamed themselves for being HIV positive. Besides, 23.4% of respondents blamed others for their condition and about 20% decided to stop childbearing for fear of transmitting the disease to their unborn children. About 11.9% of respondents feared that they might no longer have sexual partners (Table 2).

**Table 1.** Sociodemographic Characteristics of the Study Population.

Variable	Frequency (n = 387)	%
Data collection site		
DHK	336	87.3
KUSH	27	7.0
SLAK	22	5.7
Sex		
Male	103	26.8
Female	282	73.2
Age, years		
≤30	28	7.3
31-40	144	34.8
41-50	122	31.7
>50	101	26.2
Length of time living with HIV, years		
≤5	294	76.4
6-10	72	18.7
>10	19	4.9
Marital status		
Married	271	70.6
Single	69	18
Divorced	5	1.3
Widow/widower	39	10.2
Level of education		
None	4	1.0
Primary	289	75.1
Secondary	76	19.7
Tertiary	16	4.2
Number of children		
None	25	6.5
1-5	293	76.1
>5	67	17.4
Occupation		
Business	71	23.6
Farmer	343	63.1
House wife	4	1.0
Teacher	16	4.2
Others	31	8.1
Religion		
Christian	384	99.7
Muslim	1	0.3
Support group		
No	358	93
Yes	27	7.0
Any disability		
No	383	99.5
Yes	2	0.5

Abbreviations: DHK, District Hospital Kumba; KUSH, Kumba Urban Sub-Divisional Hospital; SLAK, Star Light Association Kumba.

**Table 2.** Internal Stigmatization and Fear of Respondents.

Variable	Frequency (n = 385)	%
Feel ashamed		
No	111	28.8
Yes	171	44.4
Feel guilty		
No	167	43.4
Yes	115	29.9
Blame myself		
No	159	41.3
Yes	123	31.9
Blame others		
No	192	49.9
Yes	90	23.4
Have low self-esteem		
No	266	69.1
Yes	16	4.2
Feel suicidal		
No	277	71.9
Yes	5	1.3
Decided not to attend social activities		
No	281	73.0
Yes	1	0.3
Isolated myself from my family and friends		
No	281	73
Yes	1	0.3
Decided to stop working		
No	282	73.2
Yes	2	0.52
Withdrew from education		
No	282	73.2
Yes	2	0.52
Decided not to get married		
No	264	68.6
Yes	18	4.7
Decided not to have sex		
No	265	68.8
Yes	17	4.4
Decided not to have children		
No	205	53.2
Yes	77	20
Afraid that someone would not want to become your sexual partner		
No	236	61.3
Yes	46	11.9

The external stigma percentage index score reported in the last 12 months were as follows: feeling of being gossiped (56.4%), verbal insults (19%), and an index score of 16.1. Furthermore, regarding internal stigma: feeling ashamed (57.1%), self-guilt (38.7%), self-blame (46%), blame others (29.9%), and fear of having more children (24.2%). The index score for internal stigma stood at 43.0. The total index score for external and internal stigmatization is high at 59.1 (Table 3).

**Table 3.** Categorization of Stigma Index.

Form of Stigma	% Score	Unit Value	Index
External stigma (within last 12 months)			
Avoided social gathering	1.6	20	0.3
Avoided religious events	1.0	20	0.2
Avoided family gatherings	1.0	20	0.2
Been gossiped about	56.4	20	11.3
Verbally insulted	19.0	20	3.8
Physically harassed	0.8	20	0.2
Physically assaulted	0.3	20	0.1
Total			16.1
Internal stigma (within the last 12 months)			
Feel ashamed	57.1	20	11.4
Feel guilty	38.7	20	7.7
Self-blame	46.0	20	9.2
Blame others	29.9	20	6.0
Low self-esteem	6.5	20	1.3
Feel suicidal	1.6	20	0.3
Decide not to attend social gathering	0.5	20	0.1
Isolate yourself from friends	0.8	20	0.2
Stop working	0	20	0
Stop education	0	20	0
Afraid of getting married	4.9	20	1.0
Afraid of having sex	4.9	20	1.0
Afraid of having more children	24.2	20	4.8
Total			43.0
Sum index: Internal stigma + external stigma = 43.0 + 16.1 = 59.1			

### Focus Group Discussions

The FGDs were held under the main theme “Living with HIV,” aimed at motivating participants to share their various experiences, difficulties, and coping mechanisms living with the disease. Most respondents stated that only their family members knew about their status for fear of being stigmatized by outsiders.

A woman who belonged to one support group said:

I only discovered I was HIV positive when my husband was sick and diagnosed of HIV. Everyone blamed me for being a promiscuous wife. They all said I was to be blamed. The support group was the only place I found the strength to face life.

Another lady during one of the sessions said:

I got infected after I mistakenly pierced myself with an infected syringe during my internship at a laboratory. I hid the incident from the laboratory head. I began feeling sick and weak after 6 months and decided to do an HIV test. When I first discovered I was HIV positive I attempted suicide but I was saved by my mother. I feared what people will say about me.

### Factors Affecting Stigma Level (Multivariate Analysis)

In multivariate analysis, age <30 years was significantly protective of stigma (adjusted odds ratio [AOR]: 0.39; 95% CI: 0.17-0.94, *P* = .035), while low (primary) level of education significantly increased the likelihood of stigmatization (AOR: 1.74; 95% CI: 1.02-2.97, *P* = .041; Table 4).

**Table 4.** Factors Associated with Stigmatization (Bivariate and Multivariate Analyses).

Variable	COR	95% CI	*P* Value	AOR	95% CI	*P* Value
Age, years						
<30						
≥30	0.24	0.11-0.35	.0006	0.39	0.17-0.94	.035
Level of education						
Primary						
Secondary/tertiary	2.3	1.43-3.75	.0006	1.74	1.02-2.97	.041
Length of time with HIV, years						
1-5	1.97	0.63-6.15	.2410			
6-10	1.08	0.31-3.73	.901			
11-15						
16-20	0	0-1	.973			
Marital status						
Married						
Single/divorced/widower	2.08	1.31-3.27	.0016	1.42	0.86-2.36	.170
Gender						
Female						
Male	0.056	0.33-0.94	.029	0.63	0.37-1.07	.089
Belong to a support group						
Yes						
No	0.2	0.50-2.78	.7			

Abbreviations: COR, crude odds ratios; AOR, adjusted odds ratios; CI, confidence interval.

### Testing/Diagnosis

Most cases, 57.4%, were referred to HIV treatment centers because of suspected HIV-related symptoms, and 73.2% said they received both pre- and post-HIV test counseling. Remarkably, 59.7% of them said that disclosing their status empowered them. They thought that after disclosing their status they were going to be rejected, but they were rather supported and encouraged (Table 5).

**Table 5.** Experience on Testing/Confidentiality.

Designation	Variable	Frequency (n = 385)	%
Why were you tested for HIV	During free HIV campaigns	15	3.9
	I just wanted to know	7	1.8
	Partner/family member got sick or died	8	2.1
	Partner/family member tested positive	14	3.6
	Pregnancy	15	3.9
	Preparation for marriage/sexual relationship	1	0.3
	Referred due to suspected HIV-related symptoms	221	57.4
	Referred for sexually transmitted infection	1	0.3
Did you receive counseling pre- and post-HIV counseling	Yes	282	73.2
	No	103	26.8
Has a health worker told others about your HIV status without your consent	I don’t know	2	0.5
	No	280	72.7
How confidential do you think your medical records are	I don’t know	2	0.5
	My records are confidential	280	72.7
Reactions of people when they first knew about your HIV status	Disappointed	33	8.6
	Indifferent	4	1.0
	None	10	2.6
	Supportive	196	50.9
	Very shocked	10	2.6
	Very supportive	29	7.5
Disclosure of your HIV status was an empowering experience	No	42	10.9
	Not disclosed	10	2.6
	Yes	230	59.7

## Discussion

This study elucidates the stigmatization problems faced by PLWHA and the factors associated with stigmatization in the KHD, Cameroon. The attribution of responsibility to self seems to be associated with self-stigmatization. Our findings suggest that respondents have shown feelings of guilt and shame as they consider themselves as responsible for the infection. Furthermore, the high stigma index of 59.1 found in this study is associated with the dearth of appropriate information on HIV/AIDS, particularly its mode of acquisition and spread. Our study reveals that there is a knowledge gap with people aged above 30 and those with low levels of education and especially situated in rural areas. This is very common where HIV/AIDS is still mystified and seen as a punishment from the “gods” for being promiscuous or committing a great offence. Such a belief instils feelings of shame, guilt, and self-blame which are internal stigma-related factors. Most of the older people were more affected by stigma because of the lack of perceived HIV-related stigma in older persons most often hinders educational projects targeting that age-group. This is because younger persons are usually considered to be more vulnerable. The feeling of guilt and low self-esteem among most older persons result in the latter, isolating themselves and increasing the risk of getting depressed.

Out of fear of being rejected, the minority of respondents decided not to get married. Most of them above the age of 30 did not only feel old to get married but also felt not worthy of marriage because of their HIV status. Thus, they feel unwanted and unfit for marriage. This ties in with the popular African belief whereby marriage is considered to be sacred and a symbol of purity. However, other studies have demonstrated the protective effect of strong religious believes among African Americans living with HIV and struggling with HIV-related stigma.^12^

Besides, our findings have shown that 39.5% of our participants experienced internal stigma. This result is in line with a similar study conducted in Nigeria, which showed that 63% (n = 441) of people living with HIV (PLWH) were internally stigmatized.^13^ This is attributed to most of the participants feeling ashamed because of their HIV-positive status, the feeling of low self-esteem, and self-blame for contracting the disease.

Most of the interviewees were termed “late presenters” because they had symptoms that were characteristically HIV related before the testing. The fear of finding out their status most likely prevented them from taking the test earlier. Other studies have shown that the presence of HIV symptoms was associated with stigmatization.^14^

Furthermore, most interviewees were aware that they had been the subject of gossip and some verbally insulted because of their HIV status. This is consistent with the study by Valencia-Garcia et al in Peru, where respondents declared “they would rather die than go back there for care” because of gossip from health-care professionals.^15^ The African sociocultural state is mostly based on the family, and acts of gossiping and verbal insults could instill feelings of isolation and shame. This increases self-stigma and reduces the person’s zeal to seek medical attention consequently leading to poor adherence to HAART therapy and in worst scenarios refusal to pick up their medications at treatment centers.

Most of our participants were based in rural settings where during disputes or disagreements, a person’s status could be used as a weak point to win over an argument. However, few respondents reported cases of physical harassment contrary to the findings by a similar study at the Buea Regional Hospital, where more than 10% of the participants reported cases of physical harassment.^16^

### Factors Associated with Stigmatization among Study Participants

Our findings have shown that respondents who were aged above 30 and those with a primary level of education were more likely to be stigmatized. Our study population is semiurban/rural with the greater part of the population doing subsistence farming with a primary level of education. This group of people are more likely to have misconceptions about HIV, thereby leading to self-stigmatization. They may not understand concepts regarding HIV mode of transmission, treatment, and side effects. Others may believe that being HIV positive is a death sentence and that there is no treatment for the condition.^17–19^ Furthermore, some respondents believe that HIV is a contagious disease and punishment for one’s promiscuous lifestyle. This finding is similar to a study conducted in the Congo Democratic Republic, where the level of education was significantly related to the respondent’s perceptions regarding an HIV-positive colleague.^17^

Confidentiality of participants HIV statuses has been a major concern because more than half of the respondents strongly believed that health-care providers did not disclose their HIV statuses because of their training. This is not consistent with the findings of a similar study carried out in Kenya, where more than 15% of the respondents reported that a health-care provider had disclosed their HIV status to other people without their consent.^20^

In Cameroon, there are few psychologists and social workers to cater for PLWHA. Therefore, PLWHA usually rely on support groups to obtain and share information and experiences regarding their condition. Majority of the participants in this study were not members of any support group because they were not aware of their existence or lacked the time and transport fares to do so. Data from a study carried out in Nigeria by Simpson discovered that PLWH who participated in support group activities significantly experienced less stigma than those who did not belong to support groups.^21^ Turan et al also reported that women who had not linked to HIV care after testing positive at first antenatal care visit had higher levels of depression and internalized stigma compared to women who were linked to care, therefore having less benefit for both mental and physical health.^22^

### Strengths of the Study

This is the first study in Cameroon to use the PLWHA stigma index to come out with a stigma score. This study should be an eye-opener to the type of stigma plaguing this section of the country and could be used as grounds for the measurement of the national stigma index of Cameroon.

### Study Limitation

Some participants were reluctant to tell their stories during the FGD sessions for fear of how the information will be used though they gave consent for us to do sound recording.

## Conclusion

The PLWHA in our study were highly stigmatized, experiencing more of internal stigma. Young age was protective and low level of education increased the likelihood of stigmatization. These findings serve as a key indicator in generating and implementing policies against HIV-related stigma. Older populations should be considered when drafting such policies, and focus should be on educating people on the appropriate modes and spread of HIV transmission especially in the rural communities.

## Supplemental Material

Supplemental Material, APPENDIX_I-FGD_guide - Stigmatization among People Living with HIV/AIDS at the Kumba Health District, Cameroon Click here for additional data file. Supplemental Material, APPENDIX_I-FGD_guide for Stigmatization among People Living with HIV/AIDS at the Kumba Health District, Cameroon by Thomas Obinchemti Egbe, Cynthia Adanze Nge, Hermann Ngouekam, Etienne Asonganyi and Dickson Shey Nsagha in Journal of the International Association of Providers of AIDS Care (JIAPAC)
