# Supplementary material for: Stigmatization among People Living with HIV/AIDS at the Kumba Health District, Cameroon
Source: J Int Assoc Provid AIDS Care. 2020 Jan 7;19:2325958219899305. doi: 10.1177/2325958219899305 (PMC6947670; doi:10.1177/2325958219899305)
Supplement: Supplemental Material, APPENDIX_I-FGD_guide - Stigmatization among People Living with HIV/AIDS at the Kumba Health District, Cameroon [file APPENDIX_I-FGD_guide.pdf]

APPENDIX I: Focus Group Discussion Guide (FGD guide)

This FGD was made up of 9 persons and this activity lasted for a period of 70 minutes (1 hour: 10 minutes) and followed the steps below:

1. **Welcome:** of all participants (5 minutes)
2. **Introduction:** here the purpose of the discussion shall be addressed to all the participants. Anonymity of the discussion will be assured. All participants will introduce themselves.
3. **Ground rules:** the most important rule is that only one person speaks at a time.

i. There are no right or wrong answers.

ii. If you have something to say, please feel free to kindly raise your hand and you will be called to talk. Every person's views are important and need to be heard.

iii. Questions will follow at the end.

4. Guiding questions:

1. **What is your reaction when someone mentions the name HIV/AIDS? (15 minutes)**
    - **i.** When HIV/AIDS is mentioned, what immediately comes to your head as to how the person contracted the disease?
    - **ii.** How do you see someone who is positive for HIV?
2. **How does your community view HIV? (15 minutes)**
    - **i.** What are their attitudes towards PLWHA?
    - **ii.** How do they relate with those who positive for the disease?
3. **Have you ever been discriminated against for being HIV positive or working with HIV people (for health personnel's)? (15 minutes)**

i. Do people point fingers or gossip about you because of your status or association with PLWHA?

ii. What kind of discriminative experience have you had so far?

4. **What do you wish other people knew about HIV that they currently seem to be getting wrong? (15 minutes)**

We have now reached the end of our discussion. Do you have any questions for me or any additional comments about what we have discussed so far? Thank you for taking time to talk to me today and for sharing your opinions. We truly appreciate your willingness to participate and discuss your experience with us.
